# Identification of CYP450 Family Members and Their Gonadal Expression Profiles in *Exopalaemon carinicauda*

**DOI:** 10.3390/ani16081201

**Published:** 2026-04-15

**Authors:** Shaoting Jia, Yichen Su, Yashi Hou, Kezhi Gong, Xiaotong Pan, Jianjian Lv, Jitao Li

**Affiliations:** 1 State Key Laboratory of Mariculture Biobreeding and Sustainable Goods, Yellow Sea Fisheries Research Institute, Chinese Academy of Fishery Sciences, Qingdao 266071, China; 2 Laboratory for Marine Fisheries Science and Food Production Processes, Laoshan Laboratory, Qingdao 266237, China; 3 National Experimental Teaching Demonstration Center for Aquatic Sciences, Shanghai Ocean University, Shanghai 201306, China

**Keywords:** cytochrome P450 superfamily, chromosomal localization, phylogenetic analysis, transcriptome

## Abstract

The cytochrome P450 (*CYP450*) superfamily plays important roles in a wide range of biological processes. The classification of CYP450 family members has been studied in some plants and animals; however, there are no reports on *CYP450* family members in *Exopalaemon carinicauda*. Based on publicly available whole-genome data for *E. carinicauda*, we identified 58 CYP450 family members based on the genome-wide alignment and analyzed their domains, gene structures and chromosomal locations, as well as physicochemical properties of the encoded proteins. The results revealed that CYP450 family members, widely distributed across multiple chromosomes, exhibit diverse protein properties, gene structures, and conserved motifs. Phylogenetic analysis indicated that these members are primarily clustered into subfamilies 2, 3, and 4, and the mitochondrial clan, showing close genetic relationships with homologous genes from other crustaceans. In this study, we revealed the genetic structural characteristics of the CYP450 family in *E. carinicauda*. We identified candidate genes for future research on the molecular mechanisms of *CYP450* in development and reproduction. These findings are expected to serve as a foundation for further studies in this field.

## Simple Summary

*Exopalaemon carinicauda* is an important aquaculture species that belongs to *Arthropoda*, *Crustacea*, *Decapoda*, and *Palaemonidae*. It is popular among consumers due to its delicious taste and high nutritional value. Cytochrome P450 family members play pivotal roles in various biological processes including metabolism, growth, reproduction, hormone synthesis, lipid oxidation and environmental adaptation. However, there are no reports on the CYP450 family members in *E. carinicauda*. In this study, we identified 58 CYP450 gene family members in *E. carinicauda* and investigated their expression pattern in the gonads via transcriptomic data. These findings offered potential molecular targets for elucidating the core regulatory networks underlying reproduction and development in *E. carinicauda*.

## 1. Introduction

The cytochrome P450 superfamily comprises a class of heme-containing monooxygenases that play pivotal roles in various life processes including metabolism, growth, reproduction, hormone synthesis, lipid oxidation and adaptation [1]. Recently, as omics sequencing technologies have matured, systematic studies of CYP450 genes across various species have been conducted, including studies on gene identification, functional elucidation, and evolutionary mechanisms.

In plants, CYP450 family members mainly function in metabolic pathways, especially in the functionalization of triterpene skeletons, for example, by hydroxylation and carboxylation. For instance, *CYP716A179* can mediate triterpene C-28 oxidation, participating in the biosynthesis of oleanolic acid and betulinic acid in *Glycyrrhiza uralensis* [2]. Some family members such as *CYP97* are involved in plant hormone metabolism, for example, in the synthesis of zeaxanthin and as a precursor to abscisic acid. In vertebrates, CYP450 genes also exhibit remarkable diversity. For example, 94 CYP450 genes belonging to 18 families have been identified in zebrafish [3]. *CYP1B1* participates in steroid and retinoic acid metabolism and its deficiency leads to metabolic disturbances, causing observable behavioral abnormalities in larvae and social deficits in adults. *CYP11A2* can regulate sex differentiation and gametogenesis by catalyzing pregnenolone production, while *CYP26B1/D1* is involved in hindbrain development through degrading retinoic acid. *CYP1A2* is linked to the metabolic detoxification of xenobiotics such as cadmium ions and dioxins [4]. Furthermore, *CYP21A2* deletion is associated with human congenital adrenal hyperplasia [5]. These findings underscore the functional diversity and species-specificity of CYP450 family members. Meanwhile, the functional differentiation provides important references for understanding the potential roles of CYP450 genes in other species.

CYP450 family members also exhibit diversity in crustaceans. Lafontaine et al. found that the pesticide chlordane inhibited 20-hydroxyecdysone levels and chitinase activity in *Macrobrachium rosenbergii*, suggesting the involvement of CYP450 family members in molting and environmental responses [6]. Humble et al. identified 25 CYP450 family members in *Lepeophtheirus salmonis*, among which some were linked to the development of drug resistance [7]. Nelson noted that the expansion of the CYP450 family in animals is closely related to substrate specificity differentiation through phylogenetic analysis [8]. For instance, the Halloween genes including *CYP302A1*, *CYP306A1*, *CYP307A1*, etc., are predominantly involved in ecdysteroid synthesis, while CYP3 family gene mainly functions in xenobiotic detoxification in insects and crustaceans. Research on CYP450 genes in crustaceans is limited but growing, with several genes including *CYP302a1* and *CYP330a1* having been cloned in species such as *Portunus trituberculatus*, *Carcinus maenas*, and *M. rosenbergii* [9,10,11]. The functions of *CYP307a1* and *CYP306a1* have also been examined for its roles in ecdysone regulation in *Macrobrachium nipponense* [12,13].

Recent high-throughput sequencing has enabled systematic CYP450 identification across species: 187 members in *Ginkgo biloba* [14], 48 in *Fritillaria unibracteata* [15], and 86 each in *Bombyx mori*, *Drosophila melanogaster*, and 75 in *Daphnia magna* [16,17,18], with over 60% of *B. mori* members belonging to *CYP4* and *CYP6* families. In crustaceans, 14 *CYP450* members were identified in Qinghai Gammarus lacustris [19]. However, no systematic classification of *CYP450* genes has been reported in *Exopalaemon carinicauda*.

*E. carinicauda* is an important aquaculture species [20] that belongs to arthropoda, crustacea, decapoda, and palaemonidae. It is a common polyculture species in Chinese prawn farming and holds high market value due to its delicious taste and nutritional richness. As a kind of small crustacean, it has many advantages for research, including strong adaptability, a transparent body, and a short reproductive cycle. Based on data of the *E. carinicauda* genome [21], our study aims to systematically identify CYP450 family members by integrating bioinformatics methods. Basic information on the family members including gene structures, conserved motifs, chromosomal localization, and phylogenetic characteristics is analyzed. Furthermore, by combining existing transcriptomic data from the testes and ovaries, gene expression profiles in male and female gonads are analyzed with the aim of screening key candidate genes potentially involved in reproductive development regulation. The aim of this is to provide a theoretical foundation for in-depth exploration of these genes’ molecular mechanisms in reproductive processes.

## 2. Materials and Methods

### 2.1. Ethical Statement

The research presented in this article is entirely based on genome data, transcriptome data analysis, and publicly available datasets. No experimental procedures were performed on live vertebrates or higher invertebrates. Consequently, this work falls outside the scope of regulations requiring formal ethical approval for animal experimentation.

### 2.2. Characteristics of the CYP450 Family Members

To identify the CYP450 gene family members in *E. carinicauda*, whole-genome protein sequences were retrieved from the NCBI database [21]. We aligned the sequences of CYP450 family members from multiple species, including medaka, house mouse, yellow catfish, *Drosophila melanogaster*, *Diaphanosoma celebensis*, *Cyprinus carpio*, *Penaeus vannamei*, *Homo sapiens*, *Leishmania braziliensis*. Based on the protein sequence of CYP450 family members from the reference species, the CDS sequence was extracted from the *E. carinicauda* genome using TBtools-II and subsequently translated into protein sequences. Then the BLASTP program within TBtools-II was employed to perform sequence alignment of these candidate proteins against the *E. carinicauda* genome to identify its CYP450 gene family members. A total of 58 CYP450 family members were identified in the *E. carinicauda* genome. The parameters were set as follows: an E-value threshold of 1 × 10^−5^, the BLOSUM62 scoring matrix, a word size of 3, gap penalties set to 11 for existence and 1 for extension, and a maximum of 50 target sequences. Domain analysis was conducted using the NCBI CDD database (https://www.ncbi.nlm.nih.gov/Structure/cdd, accessed on 15 May 2025), and only sequences containing a canonical *CYP450* domain were retained as final family members. The properties of the family members, including their molecular weight, and, isoelectric point, were predicted using the ExPASy ProtParam tool (https://www.expasy.org/) [22]. Finally, the signal peptides and subcellular localization were predicted using the TMHMM2.0 (https://services.healthtech.dtu.dk/services/TMHMM-2.0/, accessed on 15 May 2025) and the PSORTII online tool (https://psort.hgc.jp/form2.html, accessed on 15 May 2025) [23].

### 2.3. Phylogenetic Analysis of CYP450 Family Members in E. carinicauda

To investigate the evolutionary relationships of the CYP450 family members in *E. carinicauda*, we conducted a phylogenetic analysis. The full-length protein sequences of *CYP450* family members were downloaded from the NCBI database and combined with homologous sequences from other reference species. Sequences were aligned using ClustalX (Version 2.1) with the auto strategy. Poorly aligned regions and segments with excessive gaps either internally or at the terminals were removed by trimAI to obtain high-quality conserved blocks for phylogenetic tree construction. A maximum likelihood tree was constructed using the One Step Build a ML Tree function by TBtools-II [24], using the UltraFast BootStrap model with bootstrap analysis set to 5000 replicates to assess node support. The resulting tree was visualized and refined using the iTOL online tool. Clades were annotated and named based on evolutionary branching and the known functions of reference genes to systematically interpret the evolutionary relationships and potential functional differentiation within this gene family.

### 2.4. Analysis of Structural Characteristics of CYP450 Genes

To characterize the structures of the identified *E. carinicauda* CYP450 genes, their coding sequences were aligned with genomic sequences to determine exon–intron structures using locally installed gene structure display server software: TBtools-II. Exon–intron boundaries were analyzed to determine the number, position, and length of exons in each gene, and the distribution patterns and phases of introns were visualized with the GFF3 annotation files provided by the genome sequencing project. In addition, upstream promoter sequences of each CYP450 gene were downloaded from the genome database, and cis-acting elements were systematically scanned using the CIS-BP database and the promoter regions were defined as the 2000 bp upstream of the translation start site (ATG). Core elements related to hormone, stress, and light responses were identified to preliminarily infer the transcriptional regulatory mechanisms.

### 2.5. Transcriptome Data Sources and Analysis

Transcriptomic data of the testis and ovaries of *E. carinicauda* were obtained in our previous experiments [20] and are available from the NCBI SRA database under BioProject ID: PRJNA856985. Transcriptomic data from different ovarian developmental stages in *E. carinicauda* after eyestalk ablation [25], deposited in the NCBI database under BioProject ID: PRJNA863229, were also obtained from our prior studies. The raw data were trimmed using SeqPrep (https://github.com/jstjohn/SeqPrep, accessed on 20 July 2025), Sickle (https://github.com/najoshi/sickle, accessed on 24 July 2025) for quality control, and Trinity2.0 (http://trinityrnaseq.sourceforge.net/, accessed on 31 July 2025) for de novo assembly of clean data, and the assembled transcripts were used as the reference for subsequent expression analysis. The transcripts were annotated using BLAST2GO (http://www.blast2go.com/b2ghome, accessed on 5 August 2025). The gene expression levels were quantified using RSEM (v1.3.1) with the Trinity-assembled transcripts as reference. RPKM values were generated by RSEM, and no additional inter-sample normalization was applied beyond the built-in RPKM normalization method. Each group included three biological replicates. Differential expression analysis was performed using DESeq2 (v1.34.0) with an adjusted *p*-value (FDR) < 0.05 and |log2 fold change| ≥ 1 as the threshold for significantly differentially expressed genes. Statistical analyses were performed using GraphPad Prism 9 software and analyzed using Student’s *t*-test.

## 3. Results

### 3.1. Identification of CYP450 Gene Family Members and Protein Characterization

A total of 58 CYP450 gene family members were identified in the genome of *E. carinicauda*, the properties of which are shown in Table 1. The protein sequence lengths within the CYP450 family ranged from 129 to 892 amino acids, with molecular weights ranging from 14.59 to 100.75 kDa. The protein sequence of *CYP450_2L1_5* was the longest, consisting of 892 aa, while the protein sequence of *CYP450_3A19* was the shortest, consisting of 129 aa. The protein pI values ranged from 5.35 to 9.51, with *CYP450_4c1_1* having the lowest pI and *CYP450_302a1_2* having the highest. There were 38 proteins with an instability index greater than 40. Hydrophobicity analysis indicated that all proteins exhibited some degree of hydrophilicity, except for *CYP450_3A41_3*, which was hydrophobic.

The subcellular localization results for the CYP450 gene family members are shown in Table 2. There are four genes—*CYP450_18a1*, *CYP450_4c1_1*, *CYP450_3A24* and *CYP450_12A2*—that are localized to the nucleus. All the other members are localized to the cytoplasm.

### 3.2. Phylogenetic Analysis of CYP450 Family Members

**Figure 1:**
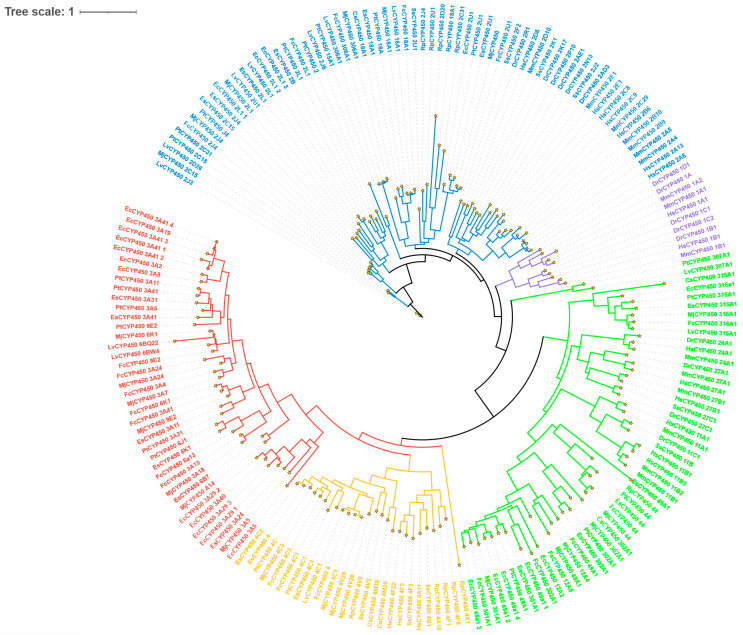
Phylogenetic tree of CYP450 family members. Different colored texts represent different clans in the figure. Purple: clan 1; blue: clan 2; red: clan 3; yellow: clan 4; green: mitochondrial clan. Species abbreviations are as follows: Hs: *Homo sapiens*; Mm: *Mus musculus*; Dr: *Danio rerio*; Cs: *Chilo suppressalis*; Dm: *Daphnia magna*; Lv: *Litopenaeus vannamei*; Fc: *Fenneropenaeus chinensis*; Pt: *Portunus trituberculatus*; Mj: *Marsupenaeus japonicus*; Rp: *Ruditapes philippinarum*; Ss: *Solea senegalensis*; Es: *Eriocheir sinensis*; Ec: *Exopalaemon carinicauda*.

The phylogenetic tree was constructed based on multiple sequence alignment of CYP450 protein sequences (Figure 1). The results indicate that the CYP450 family members are broadly clustered into five groups, corresponding to subfamilies 1, 2, 3, 4, and the mitochondrial clan. We now specify that among the five identified groups, Clan 2 contains the largest number of CYP450 genes (42 members), followed by Clan 3 (28 members) and Clan 4 (15 members). And Clan 2 and Clan 4 exhibit significant gene expansion, which may be associated with specific metabolic adaptations.

### 3.3. Gene Structure Analysis

**Figure 2:**
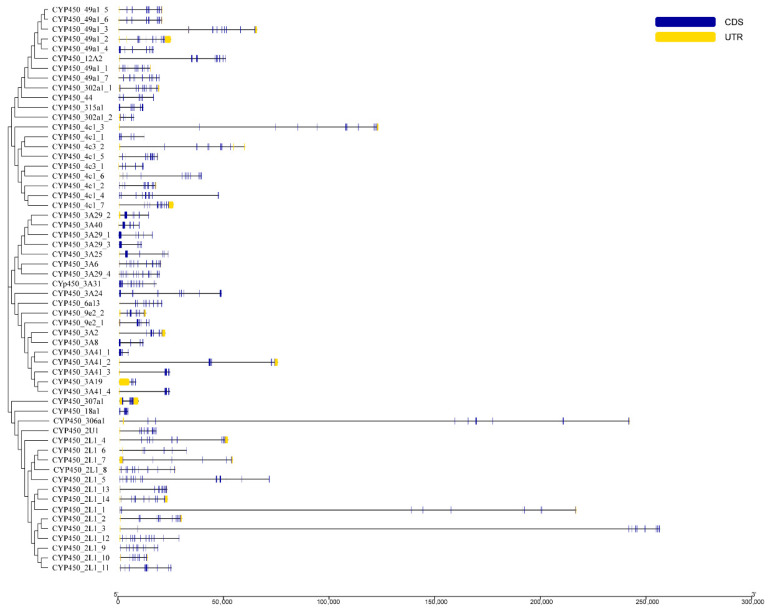
Gene structure of CYP450 family members.

The gene structures of the 58 CYP450 family members are shown in Figure 2. Their length varied, and differences were observed in exon length and number. Among the 58 CYP450 family members, *CYP450_3A29_4* consists of the most exons with 12, followed by *CYP450_3A31* and *CYP450_4c1_4*, both containing 11 exons, while *CYP450_3A19* contained the fewest exons.

### 3.4. Protein Motif and Domain Analysis

**Figure 3:**
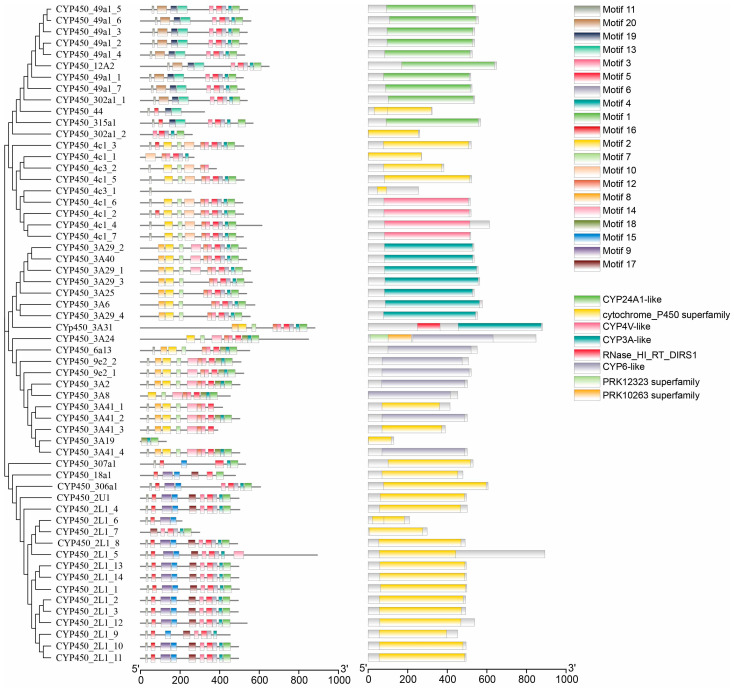
Motifs and domains of CYP450 family members.

Based on the motif analysis results (Figure 3), the CYP450 protein family could be roughly divided into three subfamilies. The order and number of motifs were similar within subfamilies but differed significantly between them. Subfamily 1 primarily consisted of nine motifs: motif 11, motif 20, motif 19, motif 13, motif 3, motif 5, motif 6, motif 4, and motif 1. Subfamily 2 primarily consisted of 10 motifs: motif 11, motif 2, motif 7, motif 10, motif 12, motif 3, motif 5, motif 6, motif 4, and motif 1. Finally, subfamily 3 primarily consisted of 9 motifs: motif 11, motif 9, motif 15, motif 17, motif 14, motif 16, motif 6, motif 4, and motif 1. The specific motif sequences are shown in Table 3.

The domain architecture analysis of CYP450 family members revealed that most contain a single conserved domain, while a few exhibit multi-domain structures. Specifically, 11 members contain only the *CYP24A1-like* domain, and 27 members possess solely the cytochrome_P450 superfamily domain. Additionally, 4, 7, and 7 members exclusively harbor *CYP4V-like*, *CYP3A-like*, and *CYP6-like* domains, respectively. In contrast, two members show complex domain compositions: *CYP450_3A31* contains both *CYP3A-like* and *RNase_HI_RT_DIRS1* domains, and *CYP450_3A24* includes *PRK12323*, *PRK10263*, and *CYP6-like* domains.

### 3.5. Chromosomal Localization Analysis

**Figure 4:**
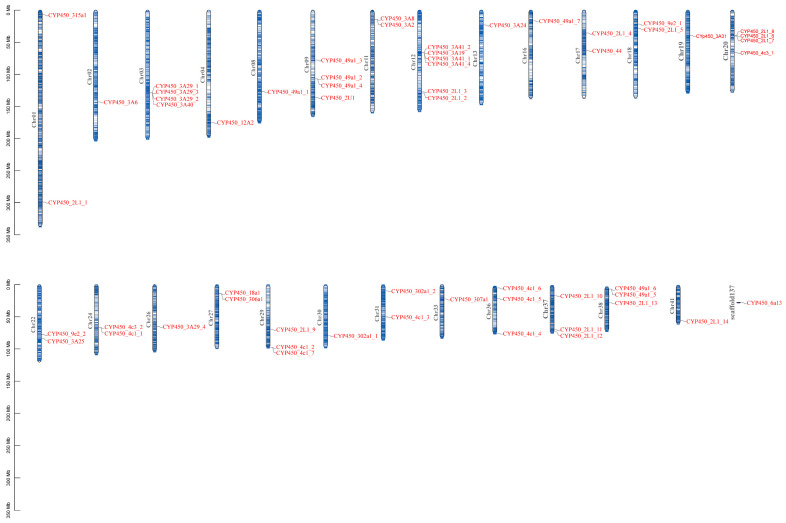
Chromosomal location of CYP450 family members. The blue vertical bars represent different chromosomes.

The chromosomal localization of 58 CYP450 family members within the genome is shown in Figure 4. The results show that these genes are widely distributed across multiple chromosomes. Specifically, the majority of the genes are scattered across chromosomes 1 to 41, while only *CYP450_6a13* is located on scaffold137. Regarding distribution density, some chromosomes harbor a relatively higher number of genes, such as Chr12 (6 genes), Chr38 (5 genes), Chr20 (4 genes), and Chr3, Chr9, and Chr31, each containing 3–4 genes. In contrast, most other chromosomes (e.g., Chr2, Chr4, Chr8) carry only 1–2 genes. This pattern indicates that the CYP450 gene family exhibits an uneven and regional distribution across the genome.

### 3.6. Differentially Expressed CYP450 Family Members Between Testes and Ovaries

**Figure 5:**
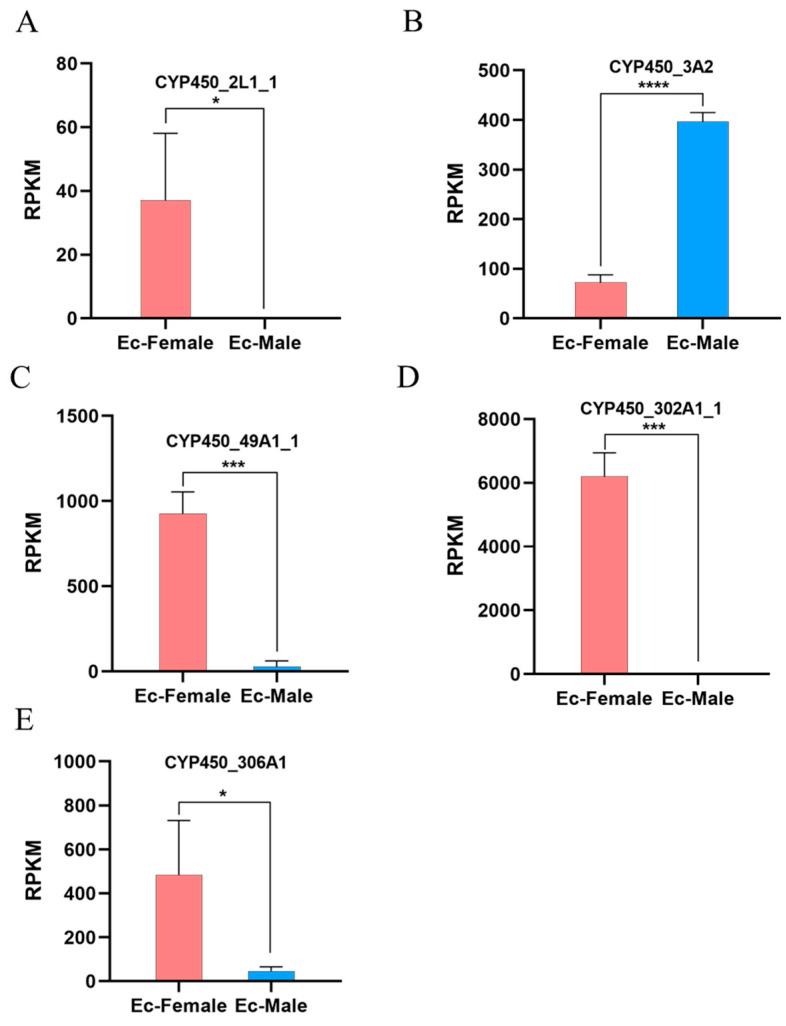
Expression of selected CYP450 family members in testes and ovaries. (**A**) The expression of *CYP450_2L1_1*; (**B**) The expression of *CYP450_3A2*; (**C**) The expression of *CYP450_49A1_1*; (**D**) The expression of *CYP450_302A1_1*; (**E**) The expression of *CYP450_305A1*. (* *p* < 0.05; *** *p* < 0.001; **** *p* < 0.0001).

To identify CYP450 family members exhibiting differential expressions, we referred to our previous transcriptome sequencing data from these organs in *E. carinicauda*. Five CYP450 family members (*CYP450_2L1_1*, *CYP450_3A2*, *CYP450_49a1_1*, *CYP450_302A1_1*, and *CYP450_306A1*) were differentially expressed between the testes and ovaries (Figure 5). Among them, *CYP450_2L1_1*, *CYP450_49a1_1*, *CYP450_302A1_1*, and *CYP450_306A1* were highly expressed in the ovaries, while *CYP450_3A2* was highly expressed in the testes. These distinct expression patterns may suggest potential sex-specific roles for these CYP450 family members in reproductive processes and gonadal development, although confirmation by qPCR is required.

### 3.7. Expression Trends of CYP450 Members After Eyestalk Ablation

**Figure 6:**
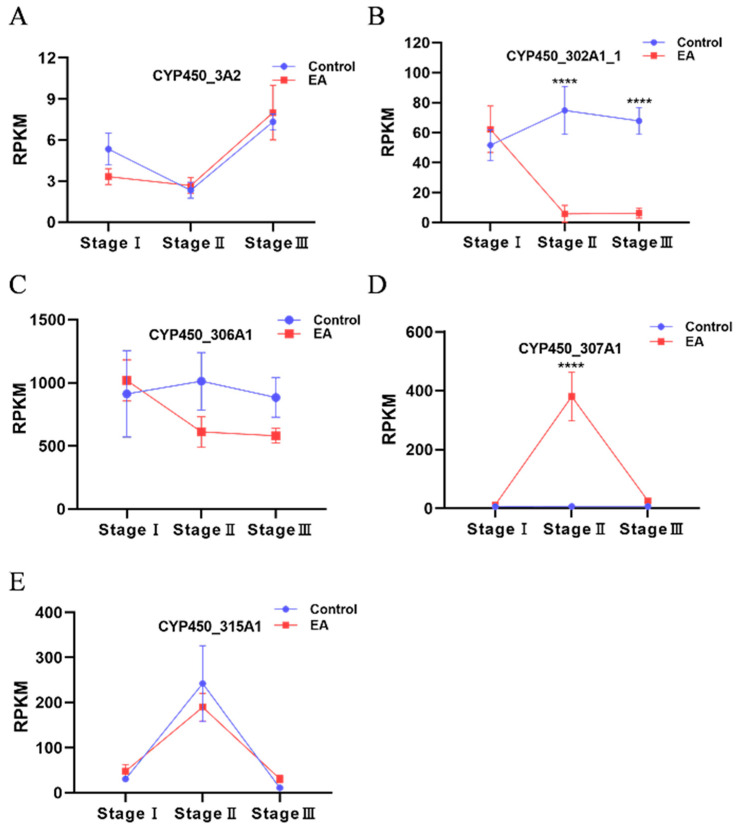
Expression trends of CYP450 family members after eyestalk ablation. Stage I: 1 day after eyestalk ablation; Stage II: 6 days after eyestalk ablation; Stage III: 11 days after eyestalk ablation. (**A**) The expression trend of *CYP450_3A2*; (**B**) The expression trend of *CYP450_302A1_1*; (**C**) The expression trend of *CYP450_306A1*; (**D**) The expression trend of *CYP450_307A1*; (**E**) The expression trend of *CYP450_315A1*. (**** *p* < 0.0001).

To examine potential involvement in hormonal regulation pathways, we referred to our previous transcriptome sequencing data from eyestalk ablation experiments. Based on this data, we found five CYP450 family members with representative expression trends (Figure 6). The expression patterns of the five CYP450 family members varied over time. Among them, *CYP450_302A1_1* and *CYP450_307A1* exhibited significant differences from the control group (*p* < 0.0001), with the former showing an initial increase followed by a slight decrease, and the latter showing a rapid increase followed by a sharp decrease. In contrast, *CYP450_3A2*, *CYP450_306A1*, and *CYP450_315A1* showed no significant differences from the control group (*p* > 0.05), although they displayed varying trends of initial increase or decrease. These expression patterns in response to eyestalk ablation suggest that *CYP450_302A1_1* and *CYP450_307A1*, might be potential involved in hormonal regulation pathways. Given that these findings are based solely on RNA-seq data, qPCR confirmation is needed in future studies.

## 4. Discussion

In this study, we identified 58 CYP450 members in *E. carinicauda* through bioinformatics analysis and constructed a phylogenetic framework, confirming the significant diversity of this gene family within crustaceans. Meanwhile, the profile of this gene family was comprehensively studied, and several specific genes such as *CYP450_307A1* and *CYP450_302A1_1*, might be linked to gonad development and the molting process through transcriptomic data analysis. This study presents molecular targets for elucidating the core regulatory networks underlying reproduction and development in *E. carinicauda*, holding significant implications for understanding the environmental adaptability of aquatic organisms.

### 4.1. The CYP450 Family in E. carinicauda Is Evolutionarily Conserved, with Significant Expansion in Specific Subfamilies

The CYP450 family members in *E. carinicauda* are evolutionarily conserved. Phylogenetic analysis revealed that CYP450 members primarily cluster into four major clades: *CYP2*, *CYP3*, *CYP4*, and the mitochondrial clan. This distribution pattern is highly conserved and resembles that reported in other crustaceans including *Macrobrachium nipponense* and *Panulirus ornatus* [26,27]. This phenomenon supports the notion that these ancient subfamilies play indispensable, fundamental roles in basic animal life processes [8]. Some family members have undergone lineage-specific expansions. For instance, the *CYP2L* subfamily showed significant expansion in *E. carinicauda*. The abundance of members within this subfamily suggests that it might have experienced gene duplication events, which is typically an evolutionary response to specific environmental pressures. Considering the ecological context of *E. carinicauda* as a coastal species frequently exposed to complex pollutant stress, this raises the hypothesis that the expansion of the *CYP2L* subfamily might have conferred enhanced detoxification capabilities for xenobiotic compounds, a possibility that requires functional validation [1].

Beyond evolutionary conservation, key differentially expressed members identified in gonads exhibit expression patterns suggestive of specialized functions. *CYP302A1*, a conserved gene in the ecdysteroid synthesis pathway in other species [11,28], was highly expressed in the ovaries. This observation suggests a potential role for this gene in reproductive regulation in crustaceans, extending its known functions beyond ecdysteroid synthesis. Conversely, *CYP3A2* was highly expressed in the testes, which may represent a species-specific adaptation in *E. carinicauda* male reproductive physiology. These distinct expression patterns indicate that while some CYP450 members retain evolutionarily conserved functions, others may have acquired specialized roles in gonadal development and reproduction.

### 4.2. CYP450 Family Members in E. carinicauda Might Play Important Roles in Crustacean Reproduction

By analyzing the specific expression patterns of the CYP450 family in *E. carinicauda* across gonads and at different developmental stages following eyestalk ablation, we provide critical clues for the family’s core functions within the crustacean reproductive regulatory network. From the expression profile analysis, we know that multiple CYP450 members, such as *CYP302A1*, are expressed highly in tissues related to reproduction, which suggests they might be involved in finely tuned reproductive regulation. In *Scylla paramamosain*, researchers found that *CYP302A1* is a key enzyme in the ecdysteroid biosynthesis pathway, catalyzing the conversion of ponasterone A to 20-hydroxyecdysone [28]. In *E. carinicauda*, the high expression of *CYP302A1* in the ovaries suggests that it might also play roles in female reproductive processes such as oocyte maturation and vitellogenesis. Similar findings have also been reported in *M. rosenbergii* [29], which may provide molecular evidence for understanding how ecdysteroids coordinate growth, development and reproduction.

In the experiment analyzing ovarian expression profiles after eyestalk ablation, *CYP307A1* expression showed a rapid response pattern—sharply increasing initially and then declining. This pattern closely aligns with the physiological processes of disinhibition from eyestalk neurohormones such as the gonad-inhibiting hormone, and the subsequent initiation of vitellogenesis. Previous studies indicate that *CYP307A1* is a rate-limiting enzyme in the early steps of ecdysteroid synthesis [30]. The observed expression changes in *CYP307A1* may reflect broader endocrine shifts resulting from eyestalk ablation. Our preliminary research also identified higher expression levels of *CYP307A1* during early ovarian development [25].

Several limitations of this study should be acknowledged. Our findings are based on transcriptomic analysis, and it is important to emphasize that mRNA expression levels are correlative in nature and do not necessarily reflect protein abundance, protein function, or enzymatic activity. While the observed expression patterns are suggestive of specific biological roles, functional validation at the protein level (e.g., Western blotting or immunohistochemistry) and direct enzyme activity assays were not performed. Therefore, these results lay the groundwork for but do not replace such validation. Future studies incorporating these approaches will be necessary to conclusively determine the functional roles of the identified CYP450 genes.

The results show that *CYP450* family members in *E. carinicauda* are far from being solely involved in detoxification. Instead, its core members are deeply embedded within the regulatory network governing the synthesis and metabolism of reproductive hormones. It is important to specifically knockdown *CYP302A1* or *CYP307A1* using methods like RNA interference, which could verify whether they indeed play roles in ovarian development and maturation. Our findings provide target genes for elucidating the molecular mechanisms of reproductive regulation in crustaceans.

## 5. Conclusions

In conclusion, our study details the genome-wide identification and characterization of CYP450 family members in *E. carinicauda*. We identified 58 CYP450 family members and located them on 27 chromosomes. Most proteins were predicted to be hydrophilic and localized in the cytoplasm, consistent with typical characteristics of CYP450 enzymes. Phylogenetic analysis indicated that the CYP450 family members of *E. carinicauda* were primarily clustered within subfamilies 2, 3, and 4, and the mitochondrial clan. Five CYP450 family members exhibited differential expression between the testes and ovaries, and several CYP450 members showed expression patterns suggesting potential roles in endocrine regulation. These findings provide a detailed description of the structural characteristics and expression patterns of the CYP450 family in *E. carinicauda*, offering candidate genes for future investigations into crustacean reproductive biology and endocrine regulation.

## Figures and Tables

**Table 1:** Protein composition and physicochemical properties of the CYP450 gene family.

Gene ID	Gene Name	Protein Length	Molecular Weight/kDa	PI	Instability Index	Grand Average of Hydropathicity	Aliphatic Index
*Chr01.g04253.m1*	*CYP450_2L1_1*	498	57.31	5.71	43.16	−0.180	89.66
*Chr12.g35711.m1*	*CYP450_2L1_2*	492	56.41	5.95	45.41	−0.145	95.67
*Chr12.g35690.m1*	*CYP450_2L1_3*	492	56.67	5.73	54.64	−0.198	93.90
*Chr17.g46067.m1*	*CYP450_2L1_4*	500	57.08	6.59	34.62	−0.185	88.36
*Chr18.g48525.m1*	*CYP450_2L1_5*	892	100.75	8.32	48.26	−0.490	80.78
*Chr20.g53478.m1*	*CYP450_2L1_6*	208	24.06	9.46	57.15	−0.023	91.88
*Chr20.g53494.m1*	*CYP450_2L1_7*	297	34.15	5.35	39.37	−0.180	85.72
*Chr20.g53477.m1*	*CYP450_2L1_8*	489	56.56	5.58	44.46	−0.151	90.72
*Chr29.g71751.m1*	*CYP450_2L1_9*	451	52.01	6.15	37.76	−0.283	88.82
*Chr37.g84839.m1*	*CYP450_2L1_10*	494	56.40	5.59	44.90	−0.199	93.72
*Chr37.g86113.m1*	*CYP450_2L1_11*	494	57.16	6.78	41.84	−0.202	89.39
*Chr37.g86193.m1*	*CYP450_2L1_12*	537	61.83	6.51	39.56	−0.298	87.19
*Chr38.g86722.m1*	*CYP450_2L1_13*	495	56.69	6.69	35.81	−0.173	96.30
*Chr41.g91361.m1*	*CYP450_2L1_14*	495	57.07	6.36	35.91	−0.208	88.22
*Chr09.g28246.m1*	*CYP450_2U1*	496	57.66	7.77	39.05	−0.292	87.00
*Chr27.g67194.m1*	*CYP450_306A1*	605	69.37	5.83	44.47	−0.294	90.41
*Chr33.g78411.m1*	*CYP450_307A1*	529	60.38	6.30	40.00	−0.261	85.29
*Chr27.g67191.m1*	*CYP450_18A1*	478	54.00	9.37	52.48	−0.316	79.14
*Chr11.g31708.m1*	*CYP450_3A2*	501	57.02	6.40	45.28	−0.091	87.64
*Chr02.g07043.m1*	*CYP450_3A6*	576	65.30	8.82	31.93	−0.214	87.57
*Chr11.g31707.m1*	*CYP450_3A8*	452	51.73	5.50	48.44	−0.120	87.59
*Chr12.g34874.m1*	*CYP450_3A19*	129	14.59	5.91	33.78	−0.118	97.52
*Chr13.g36729.m1*	*CYP450_3A24*	847	92.88	6.73	61.84	−0.316	79.98
*Chr22.g58734.m1*	*CYP450_3A25*	535	61.29	6.40	38.51	−0.105	94.06
*Chr03.g10506.m1*	*CYP450_3A29_1*	555	63.90	8.01	34.01	−0.157	96.45
*Chr03.g10508.m1*	*CYP450_3A29_2*	534	61.02	8.72	35.91	−0.094	96.93
*Chr03.g10507.m1*	*CYP450_3A29_3*	563	64.38	8.86	37.47	−0.162	91.58
*Chr26.g66158.m1*	*CYP450_3A29_4*	552	62.77	8.80	44.62	−0.231	93.04
*Chr19.g50915.m1*	*CYP450_3A31*	880	98.48	6.16	47.50	−0.389	83.07
*Chr03.g10509.m1*	*CYP450_3A40*	535	61.56	8.55	36.49	−0.101	99.98
*Chr12.g34876.m1*	*CYP450_3A41_1*	413	47.03	9.03	43.09	−0.101	85.71
*Chr12.g34871.m1*	*CYP450_3A41_2*	500	56.87	8.38	40.29	−0.089	89.72
*Chr12.g34879.m1*	*CYP450_3A41_3*	389	43.99	8.04	41.38	0.027	92.52
*Chr12.g34879.m1*	*CYP450_3A41_4*	500	56.84	7.53	39.89	−0.102	88.96
*scaffold137.g98188.m1*	*CYP450_6A13*	550	61.91	8.87	48.07	−0.008	94.71
*Chr18.g48378.m1*	*CYP450_9E2_1*	520	59.35	8.57	42.29	−0.156	88.69
*Chr22.g58666.m1*	*CYP450_9E2_2*	506	58.28	6.45	32.47	−0.108	84.01
*Chr24.g62201.m1*	*CYP450_4C1_1*	269	31.07	5.35	40.98	−0.499	86.69
*Chr29.g72426.m1*	*CYP450_4C1_2*	519	59.77	6.88	42.98	−0.250	93.53
*Chr31.g75635.m1*	*CYP450_4C1_3*	520	60.24	6.36	39.07	−0.399	86.98
*Chr36.g84414.m2*	*CYP450_4C1_4*	612	70.35	7.23	43.98	−0.418	88.09
*Chr36.g83447.m1*	*CYP450_4C1_5*	522	60.35	8.65	36.65	−0.227	92.26
*Chr36.g83037.m1*	*CYP450_4C1_6*	515	59.31	7.58	43.01	−0.230	96.78
*Chr29.g72429.m1*	*CYP450_4C1_7*	519	59.67	7.16	39.28	−0.217	95.39
*Chr20.g53902.m1*	*CYP450_4C3_1*	254	29.57	9.43	51.40	−0.116	96.77
*Chr24.g62199.m1*	*CYP450_4C3_2*	382	44.42	6.18	35.60	−0.292	86.75
*Chr04.g14492.m1*	*CYP450_12A2*	648	74.31	9.18	54.47	−0.451	83.81
*Chr17.g46655.m1*	*CYP450_44*	322	36.84	6.57	44.50	−0.182	92.30
*Chr08.g25434.m1*	*CYP450_49A1_1*	518	59.58	9.38	40.03	−0.333	87.70
*Chr09.g27757.m1*	*CYP450_49A1_2*	537	61.34	8.67	41.46	−0.246	91.25
*Chr09.g27435.m1*	*CYP450_49A1_3*	538	61.55	8.96	49.34	−0.382	81.34
*Chr09.g27758.m1*	*CYP450_49A1_4*	525	60.05	8.89	43.54	−0.221	93.92
*Chr38.g86333.m1*	*CYP450_49A1_5*	540	62.20	8.62	44.34	−0.384	79.46
*Chr38.g86332.m1*	*CYP450_49A1_6*	556	64.13	8.57	48.54	−0.365	78.72
*Chr16.g43710.m1*	*CYP450_49A5_7*	524	60.11	8.82	45.51	−0.390	85.04
*Chr30.g74098.m2*	*CYP450_302A1_1*	538	61.13	8.72	45.30	−0.321	91.51
*Chr31.g74724.m1*	*CYP450_302A1_2*	260	29.13	9.51	57.62	−0.079	95.69
*Chr01.g00149.m1*	*CYP450_315A1*	567	63.29	9.28	49.58	−0.322	83.05

**Table 2:** The subcellular localization of CYP450 family members.

Gene ID	Localization
*CYP450_18A1*, *CYP450_4C1_1*, *CYP450_3A24*, *CYP450_12A2*	Nucleus
*CYP450_2L1_2*, *CYP450_2L1_3*, *CYP450_2L1_6*, *CYP450_2L1_7*, *CYP450_2L1_8*, *CYP450_2L1_9*, *CYP450_2L1_10*, *CYP450_2L1_11*, *CYP450_3A31*, *CYP450_2L1_14*, *CYP450_9E2_1*, *CYP450_3A8*, *CYP450_3A19*, *CYP450_44*, *CYP450_49A1_1*, *CYP450_49A1_2*, *CYP450_49A1_3*, *CYP450_49A1_4*, *CYP450_49A1_5*, *CYP450_49A1_6*, *CYP450_49A5_7*, *CYP450_302A1_1*, *CYP450_302A1_2*, *CYP450_315A1*	Cytoplasm
*CYP450_2L1_1*, *CYP450_2L1_4*, *CYP450_2L1_5*, *CYP450_2L1_12*, *CYP450_2L1_13*, *CYP450_2U1*, *CYP450_306A1*, *CYP450_307A1*, *CYP450_3A2*, *CYP450_3A6*, *CYP450_3A25*, *CYP450_3A29_1*, *CYP450_3A29_2*, *CYP450_3A29_3*, *CYP450_3A29_4*, *CYP450_3A40*, *CYP450_3A41_1*, *CYP450_3A41_2*, *CYP450_3A41_3*, *CYP450_3A41_4*, *CYP450_6A13*, *CYP450_9E2_2*, *CYP450_4C1_2*, *CYP450_4C1_3*, *CYP450_4C1_4*, *CYP450_4C1_5*, *CYP450_4C1_6*, *CYP450_4C1_7*, *CYP450_4C3_1*, *CYP450_4C3_2*,	Cytomembrane

**Table 3:** Protein motif information of CYP450 family members.

Motif	Protein Sequence	No. of AA
Motif 1	NKKDIHPYAYLPFGAGPRNCIGERFARMELKIFLARL	37
Motif 2	DPWMNTMLLNLRGQKWKSVRSLLTPTFSSGKMKDMFHLVNE	41
Motif 3	DTTSTTLAWTLYLLAKHPEIQ	21
Motif 4	DPKYWPDPEEFDPERFL	17
Motif 5	GPLTYEDLMELKYLEAVIKEVLRLYPPVP	29
Motif 6	GVGRELTEDTVLGGYRIPKGT	21
Motif 7	TLDVICECAFGLECNAQRDEN	21
Motif 8	YMGLKPFLVVCDPELIRQILIKDFDHFTNRP	31
Motif 9	EAGIIGSNGDVWVNNRRFALRHLRDLGMGKSSLEEAIQEEARILVEDFKK	50
Motif 10	QQRQARPWLQPDILFKLLGYAKEHDACLKVLHDMSYKCIRERRKQYQERK	50
Motif 11	PGPPGLPILGS	11
Motif 12	KKVLTDEEIIANVDLFMLAGY	21
Motif 13	MDQVTLEFMERISSFQDEHGEMPEDFQVELYKWALESVSLVALNRRLGCL	50
Motif 14	GLSLFPKETQFFKNVVEETLAARRKGTKRGDFLDLLLEAQSGEDLGDPSK	50
Motif 15	GKPVEIPWSLNVAVLNVIWKMVAGKRYDM	29
Motif 16	KYGDIFSWKLGGQIFVFICDY	21
Motif 17	EIIDEHKKNFDPDNPKDYIDAYLIEMKKKTNEAGS	35
Motif 18	CTKDYKIPGTNLTVPKGLSVQVPVYSIHH	29
Motif 19	GLLIENGEEWKRVRSRVQTPMMKPKNVNAYL	31
Motif 20	HKFWRKMVEEYGPIVRLDMPGMPPLVFITDPEDCEMLVRSTMDNPTRPG	49

## Data Availability

The original contributions presented in this study are included in the article. Further inquiries can be directed to the corresponding author.
